# p53-dependent DNA repair during the DNA damage response requires actin nucleation by JMY

**DOI:** 10.1038/s41418-023-01170-9

**Published:** 2023-05-04

**Authors:** Ignacio Rodriguez-Pastrana, Eleni Birli, Amanda S. Coutts

**Affiliations:** 1 School of Science and Technology, Department of Biosciences, Nottingham Trent University, Clifton Lane, Nottingham, NG11 8NS UK; 2 John van Geest Cancer Research Centre, Nottingham Trent University, Clifton Lane, Nottingham, NG11 8NS UK

**Keywords:** Tumour-suppressor proteins, Cell biology, Molecular biology, RNA

## Abstract

The tumour suppressor p53 is a nuclear transcription factor with key roles during DNA damage to enable a variety of cellular responses including cell cycle arrest, apoptosis and DNA repair. JMY is an actin nucleator and DNA damage-responsive protein whose sub-cellular localisation is responsive to stress and during DNA damage JMY undergoes nuclear accumulation. To gain an understanding of the wider role for nuclear JMY in transcriptional regulation, we performed transcriptomics to identify JMY-mediated changes in gene expression during the DNA damage response. We show that JMY is required for effective regulation of key p53 target genes involved in DNA repair, including *XPC*, *XRCC5* (Ku80) and *TP53I3* (PIG3). Moreover, JMY depletion or knockout leads to increased DNA damage and nuclear JMY requires its Arp2/3-dependent actin nucleation function to promote the clearance of DNA lesions. In human patient samples a lack of JMY is associated with increased tumour mutation count and in cells results in reduced cell survival and increased sensitivity to DNA damage response kinase inhibition. Collectively, we demonstrate that JMY enables p53-dependent DNA repair under genotoxic stress and suggest a role for actin in JMY nuclear activity during the DNA damage response.

## Introduction

The human tumour suppressor p53 plays a crucial role in the cellular response to a variety of stressors including DNA damaging and chemotherapeutic agents [1]. As a transcriptional regulator p53 alters gene expression programmes that ultimately impact on cellular outcome that can include cell cycle arrest, apoptosis or DNA repair [2]. Although we still have an incomplete understanding of the mechanisms involved in p53 regulation and how this influences tumour suppression research clearly supports the coordination of DNA repair in being critical for tumour suppression mediated by p53 (reviewed in [3]). Genotoxic stressors (*e*.*g*., DNA damaging and chemotherapeutic agents) trigger a myriad of tightly coordinated responses during the DNA damage response (DDR) primarily via ATM, ATR and DNA-PK, members of the phosphatidylinositol 3-kinase-related kinases (PIKK) family [4]. When activated, these kinases phosphorylate multiple targets including p53 [5, 6] and H2AX [7, 8] leading to DNA repair, cell cycle arrest or apoptosis [4]. p53 plays a direct role in DNA repair during the DDR through transcriptional activation of genes involved in a range of DNA repair pathways including for example, *TP53I3* (PIG3), *XRCC5* (Ku80) and *XPC* (reviewed in [9]). Defects in p53 function can lead to reduced DNA repair resulting in genomic instability and ultimately tumour development [10]. Interestingly, tumour cells commonly present defects or reduced expression in DNA repair genes which causes the loss of one or more DDR pathways; thus providing the molecular rationale for the use of small-molecule inhibitors of the DDR in cancer therapeutics to exploit these vulnerabilities [10, 11].

The p53 response to DNA damage is influenced by a variety of cofactors that positively and negatively regulate p53 activity [12, 13]. JMY (junction-mediating and regulatory protein) is an actin nucleator and DNA damage-responsive protein. JMY is a member of the WASp (Wiskott–Aldrich Syndrome protein) family of actin nucleation-promoting factors (NPFs) that regulate filamentous (F) actin formation by activating the actin-related protein 2/3 (Arp2/3) complex [14, 15], although JMY uniquely nucleates actin in both an Arp2/3-dependent and independent fashion [15]. JMY localises to both the cytoplasm and the nucleus where cytoplasmic JMY regulates the formation of actin filaments promoting cell motility and invasion [14, 15]. Interestingly, cytoplasmic JMY also increases survival in cells undergoing autophagy, during which JMY’s actin nucleation activity enhances the formation and maturation of the autophagosomes [16]. In response to genotoxic stress, JMY undergoes nuclear accumulation where it has been shown to enhance p53-dependent transcriptional activation of *Bax* [14, 17]. It is now clear that many of the key players that control actin nucleation in the cytoplasm can also be found in the nucleus and actin plays key roles in nuclear events such as DNA repair [18, 19] and transcriptional regulation [20, 21]. However, we still need to fully understand the impacts of actin nucleators during the DDR and importantly how this might impact on p53 activity in human cancer.

Here, we report that nuclear JMY promotes DNA repair and cell survival during the DDR by enhancing the expression of p53 transcriptional targets involved in DNA repair. JMY-deficient cells exhibit increased DNA damage accumulation and impaired DDR signalling. Importantly, nuclear JMY requires Arp2/3-dependent actin nucleation activity for the efficient clearance of DNA strand breaks. The lack of JMY ultimately increases sensitivity to DNA-damaging agents and, in particular, sensitises cells to DDR inhibition leading to reduced cell survival. Collectively, our results indicate that JMY enhances p53 transcriptional activity to efficiently repair DNA breaks under genotoxic stress and suggest a role of actin nucleation in JMY activity during the DDR.

## Results

### JMY influences the expression of p53 target genes involved in DNA repair

**Fig. 1:**
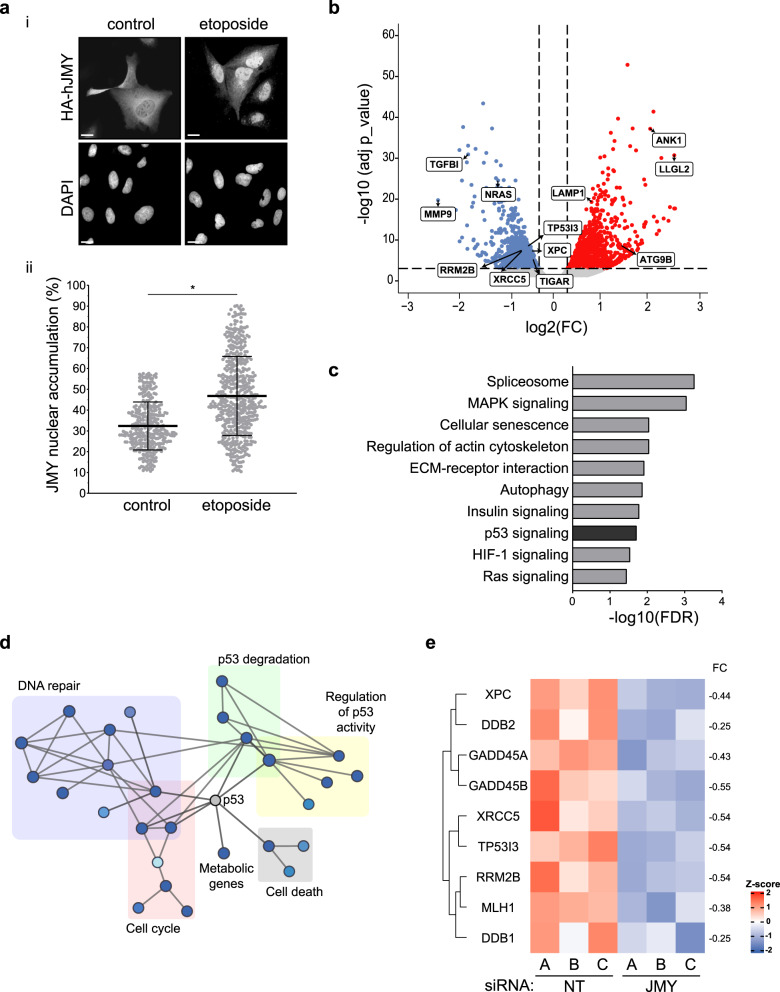
JMY impacts p53-dependent gene expression. **a** (i) U2OS cells expressing HA-tagged wild-type human JMY (HA-hJMY) were treated with DMSO vehicle (control) or 50 µM etoposide for 6 h. JMY was detected using anti-HA and DAPI was used to visualise nuclei. Scale bar = 10 µm. (ii) Quantification of JMY nuclear versus cytoplasmic accumulation (mean ± SD), N = ≥ 300 cells per treatment, * p < 0.0001, Mann-Whitney test. **b** Volcano plot represents differentially expressed genes influenced by JMY (q-value < 0.001). Red = upregulated, blue = downregulated and grey = not significant. **c** Selected enriched KEGG pathways. The threshold was set as Benjamini-Hochberg FDR < 0.05. **d** Enrichment map representation of p53-related and DNA repair pathways from Reactome database, FDR < 0.05. Nodes and clusters were manually arranged for clarity. **e** Heatmap showing the relative expression of top p53-downstream targets involved in DNA repair. Changes in gene expression levels are represented as log_2_(FC). Red = upregulated, blue = downregulated. FDR False Discovery Rate, FC Fold-change expression.

To understand the impact of JMY on gene expression during the DNA damage response we performed transcriptomic analysis (RNA-sequencing) in U2OS osteosarcoma cells under etoposide treatment where JMY undergoes significant nuclear accumulation (Fig. 1ai,ii; [14]). We compared cells treated with either non-targeting or JMY siRNA to identify JMY-mediated impacts on gene expression (Fig. 1b; Fig. S1a, b). Pathway enrichment analyses identified cellular processes that were significantly enriched including the p53 signalling response (q-value = 0.019; Fig. 1c; SI Table 1). JMY depletion resulted in reduced expression of the p53 targets *BAX* and *CDKN1A* in support of previous studies [14, 17], but our transcriptomic analysis also identified p53 targets such as *Puma*/*Bbc3* and *TIGAR* that underwent reduced expression upon JMY depletion (SI Table 2). Closer inspection revealed that while JMY influenced the expression of a range of p53 targets there was enrichment of genes involved in DNA repair with the vast majority of these targets being significantly down-regulated with JMY depletion (Fig. 1d,e; SI Table 1).

**Fig. 2:**
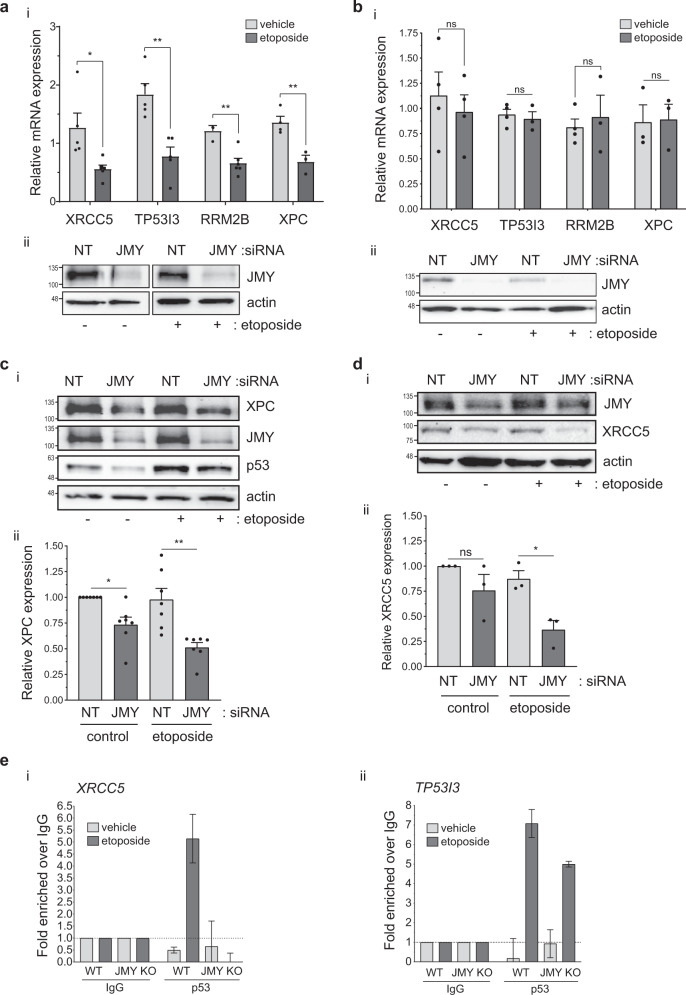
JMY influences the expression of DNA repair genes. U2OS (**a**) and Saos2 (**b**) cells were transfected with JMY or non-targeting siRNA for 72 h and treated with vehicle (-, DMSO) or etoposide (+, 50 µM) for the last 6 h before harvesting. (i) Changes in gene expression are present as fold mRNA expression relative to non-targeting controls after normalising with GAPDH (mean ± s.e.m.). *n* = > 3 independent experiments. (ii) Western blot represents JMY knockdown. U2OS cells transfected and treated as in **a** before protein extraction. (i) Western blots represent XPC (**c**), XRCC5 (**d**) levels. (ii) Graph represents expression levels after normalising for actin (mean ± s.e.m.). *n* = 3–7 independent experiments. **e** HAP1 parental (WT) and JMY knockout (JMY KO) cells were treated with vehicle (control) or etoposide (500 nM) for 6 h before ChIP. qPCR was performed on ChIP chromatin and results are expressed as fold over IgG (mouse non-specific IgG) after normalising to input levels showing p53 recruitment to *XRCC5* (i) or *TP53I3* (ii) promoters. *n* = 2 independent experiments, fold ± SD. ns: not significant, *p < 0.05 and **p < 0.01, Student’s *t*-test.

We validated JMY-dependent regulation of p53 targets involved in DNA repair including *XRCC5* and *XPC* by qPCR, confirming that JMY deficiency resulted in a significant reduction in expression during etoposide treatment (Fig. 2a). Conversely, in p53-null Saos2 osteosarcoma cells JMY depletion had little impact on expression (Fig. 2b), confirming that JMY enhances p53-dependent transcription during etoposide-mediated DNA damage. This was reflected in changes to protein expression as siRNA-mediated JMY depletion in U2OS cells resulted in significant reductions in both XPC and XRCC5 (Ku80) protein levels (Fig. 2c, d). The impact of JMY on p53-dependent gene expression was not restricted to a single cell type as we also obtained similar results in p53 wild-type MCF7 breast cancer cells (Fig. S1c). Nor was the effect restricted to etoposide since JMY also undergoes significant nuclear localisation in U2OS cells during treatment with the ultraviolet-radiation mimetic 4NQO (Fig. S2a) and under these conditions JMY depletion also resulted in a reduction in XPC and XRCC5 (Fig. S2b, c).

Importantly, we compared HAP1 chronic myelogenous leukaemia-derived parental (wild-type p53) and JMY knockout cells (Fig. S3ai), where ablation of *JMY* resulted in a decrease in both XPC and XRCC5 mRNA and protein (Fig. S3aii, b, c). To determine if JMY influenced p53 recruitment to target genes we performed chromatin-immunoprecipitation (ChIP) in the HAP1 cell lines to allow the comparison in the presence and absence of JMY. In JMY knockout cells there was a marked reduction in p53 recruitment to target genes under etoposide treatment (Fig. 2ei,ii, S3di, ii). Together this demonstrates that nuclear JMY enhances p53 transcriptional activity and recruitment to target genes during the DNA damage response and in particular positively influences the expression of genes involved in DNA repair.

### JMY deficiency results in increased DNA damage

**Fig. 3:**
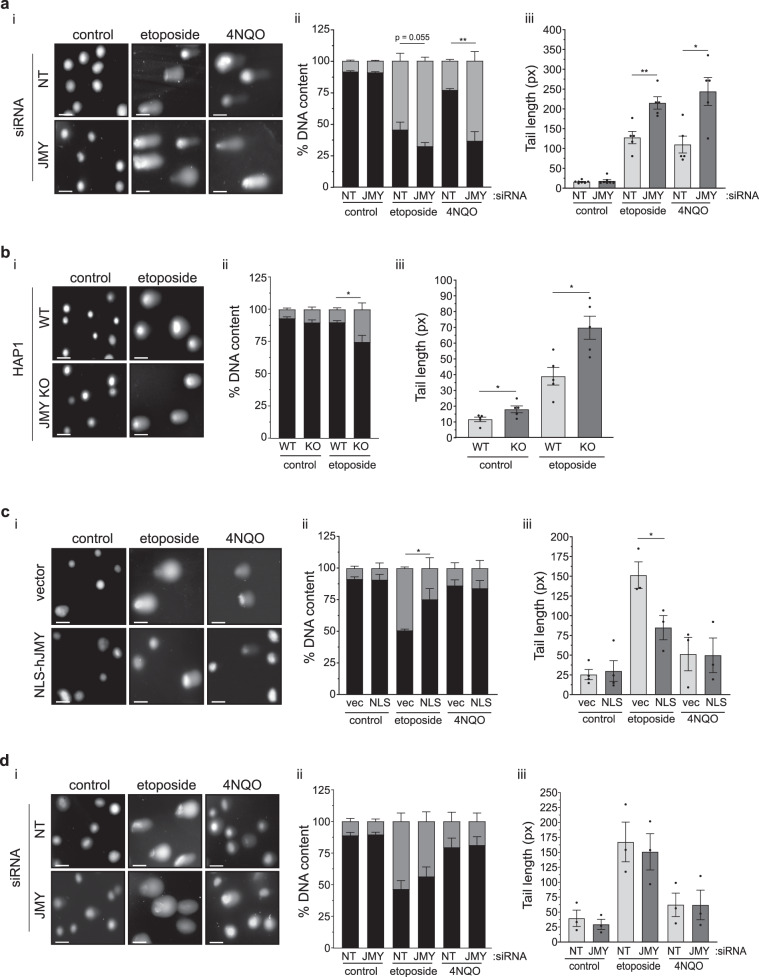
Nuclear JMY reduces DNA damage. **a** (i) U2OS cells transfected with JMY or non-targeting (NT) siRNA for 72 h before treating with vehicle (control), etoposide (10 µM) or 4NQO (100 nM) for the last 16 h. Comets were stained with Hoechst-33342. (ii) Quantification of the DNA content distributed between the head (black) and tail (grey) of the comet (ii) and the comet tail length (iii), *n* = 5 independent experiments (mean ± s.e.m.). **b** HAP1 parental (WT) and JMY knockout (JMY KO) cells were treated with vehicle (control) or etoposide (500 nM) for 16 h. Comet DNA content (ii) and tail length (iii) were calculated as in **a**, *n* = 5 independent experiments (mean ± s.e.m.). **c** i U2OS cells expressing FLAG-NLS-hJMY (NLS-hJMY, NLS) or vector control (vector) were treated as in **a**. Comet DNA content (ii) and tail length (iii) were calculated as in **a**, *n* = 3 independent experiments (mean ± s.e.m.). **d** Saos2 cells were transfected and treated as in **a**. Comet DNA content (ii) and tail length (iii) were calculated as in **a**, *n* = 3 independent experiments (mean ± s.e.m.). Scale bar = 40 µm. ns: not significant, *p < 0.05, **p < 0.01, Student’s *t*-test.

The fact that JMY deficiency significantly reduced the expression of key p53 target genes involved in DNA repair suggested that JMY may impact on the accumulation of DNA damage. To measure JMY-mediated impacts on DNA strand breaks we employed alkaline comet assays to directly measure DNA damage in single cells [22]. As expected, both etoposide and 4NQO treatment resulted in a significant accumulation of DNA strand breaks as inferred by comet tail DNA content and length after 16 h (Fig. 3a). While JMY depletion had little impact on the amount of detectable DNA damage under control non-perturbed conditions, under both etoposide and 4NQO treatment JMY depletion resulted in a marked increase in the amount of DNA damage detected (Fig. 3a). These results were recapitulated in both JMY siRNA treated MCF7 (Fig. S4a) and in JMY knockout HAP1 (Fig. 3b) cells. We reasoned that an absence of nuclear JMY during the DDR was negatively impacting on the cells ability to repair DNA and, therefore, overexpressing nuclear JMY should have the converse effect. To test this we used U2OS cells stably overexpressing nuclear localised human JMY (Fig. S4b) to demonstrate that nuclear JMY expression reduced the accumulation of DNA damage during the DDR (Fig. 3c). Moreover, in the absence of p53, JMY depletion did not result in increased DNA damage accumulation (Fig. 3d). In all our results suggest that nuclear JMY is able to enhance p53-dependent DNA repair during genotoxic stress.

**Fig. 4:**
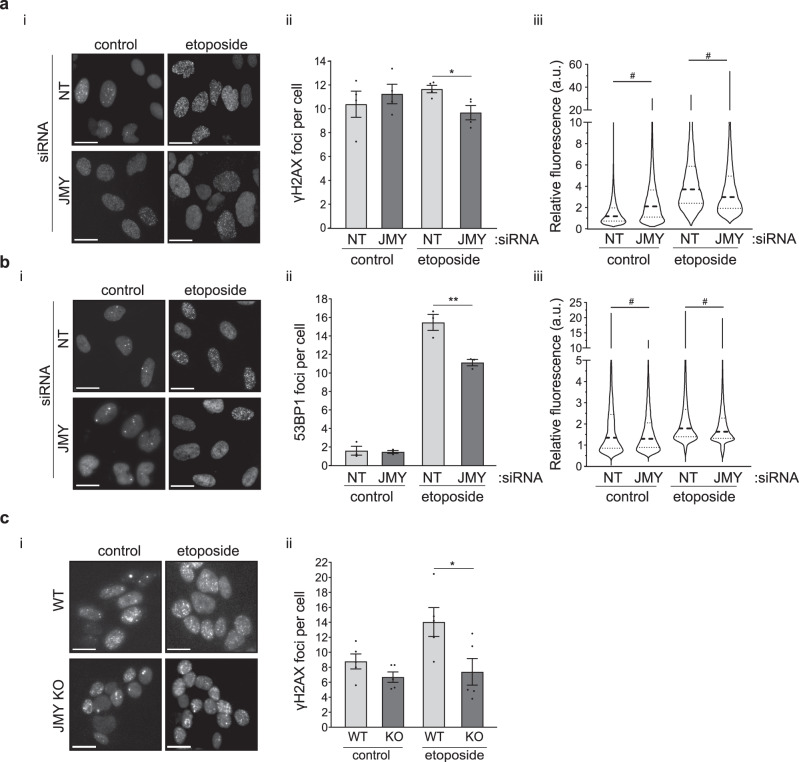
DDR signalling is impacted by JMY. **a**, **b** U2OS cells transfected with JMY or non-targeting siRNA for 72 h and treated with vehicle (control) or etoposide (50 µM) for the last 6 h before immunofluorescence. Foci were detected with anti-γH2AX (**a**) or anti-53BP1 antibodies (**b**). (ii) Graphs represent the mean number of foci per cell ± s.e.m. for γH2AX (**a**) or 53BP1 (**b**), *n* = 3-4 independent experiments each with *N* = > 100 cells per condition. (iii) Violin plots represent relative fluorescence intensity for γH2AX (**a**) or 53BP1 (**b**) (median and quartiles) *N* = > 300 cells per condition pooled from *n* = 3-4 independent experiments. **c** (i) HAP1 parental (WT) and JMY knockout (JMY KO) cells were treated with either vehicle (control) or etoposide (500 nM) for 6 h before performing immunofluoresence. (ii) Quantification of γH2AX foci per cell (mean ± s.e.m.), *n* = 5 independent experiments. Scale bars = 10 µm. *p < 0.05 and **p < 0.01, Student’s *t*-test. #*p* < 0.0001, Mann-Whitney U test.

DNA strand breaks induce the formation of DNA damage response foci via the recruitment of repair proteins and these are commonly characterised by markers such as phosphorylated histone H2AX (γH2AX) and 53BP1 [23]. JMY depletion reduced the number of γH2AX and 53BP1 foci detected by immunofluorescence during the DDR (Fig. 4a, b; S5a), as well as decreased total cellular γH2AX levels detected by Western blotting (Fig. S5b). This was also recapitulated in JMY knockout cells where we observed a marked reduction in both γH2AX foci and total levels during the DDR (Fig. 4c; S5c). This suggests that a lack of JMY compromises the cellular response to DNA damage. In support of this, we observed a decrease in total cellular ATM and ATR activity upon JMY depletion or knockout during etoposide treatment (Fig. S5d, e); thus suggesting that JMY enables efficient DDR signalling.

### JMY-mediated actin nucleation enhances DNA repair

**Fig. 5:**
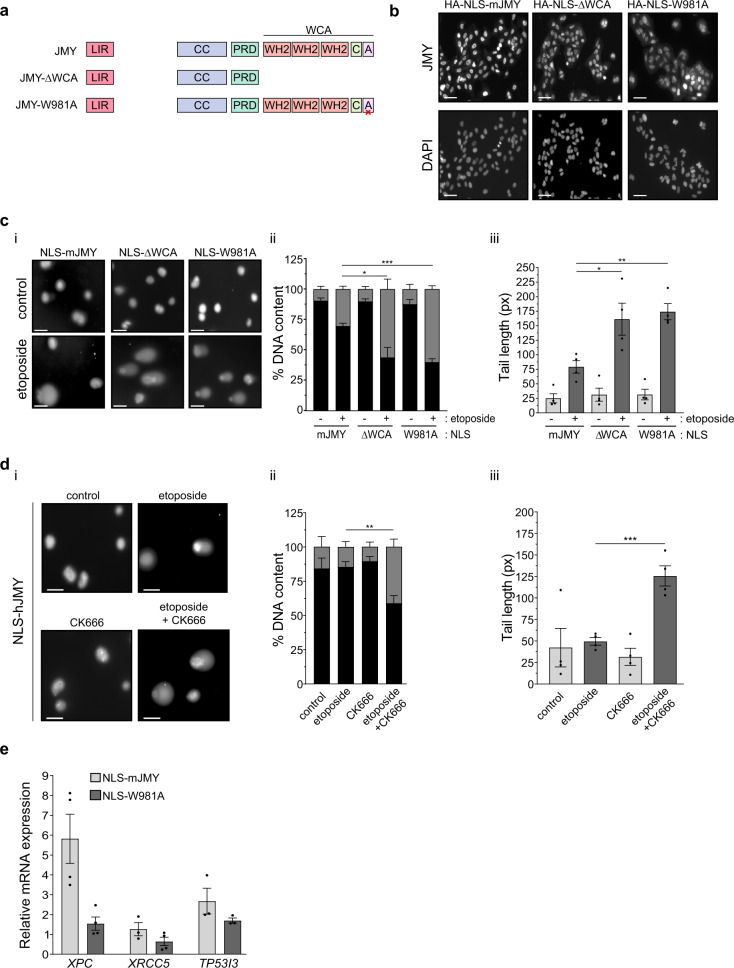
JMY-mediated actin nucleation reduces DNA damage. **a** Schematic representation of JMY derivatives lacking the WCA actin nucleation domain (ΔWCA) or presenting a single mutation compromising Arp2/3-dependent actin nucleation (W981A). **b** Immunofluorescence of U2OS cells stably expressing nuclear JMY and derivatives detected using anti-HA antibody. **c** U2OS stable cell lines were treated with either vehicle (control) or etoposide (10 µM) for 16 h. Graphs represent mean ± s.e.m. of comet DNA content distributed between the head (black) and tail (grey) (ii) and comets’ tail length (iii), *n* = 4 independent experiments. **d** U2OS NLS-hJMY stable cells were treated with either vehicle (control), etoposide (10 µM), and CK666 (100μM) as indicated for 16 h. Comet DNA content (ii) and tail length (iii) were calculated as in **c**, *n* = 4 independent experiments (mean ± s.e.m.) **e** U2OS stable JMY cell lines were treated with etoposide (50 μM) for 6 h before RNA isolation and RT-qPCR. Results represent fold mRNA expression relative to vehicle treatment after normalising with *GAPDH* (mean ± s.e.m.). *n* = 4 independent experiments. Scale bars = 40 µm. *p < 0.05, **p < 0.01 and ***p < 0.001 Student’s *t*-test.

Because nuclear actin is known to play roles in both DNA repair and transcriptional regulation [20] and previous work suggested that actin nucleation may play a role in JMY’s nuclear activities [14], we considered whether JMY’s actin nucleation ability influenced JMY-mediated DNA repair. To address this we used stable cell lines to enable a comparison between nuclear JMY with and without its Arp2/3-dependent and independent actin nucleation activity ([14], Fig. 5a, b, Fig. S6a, b). As observed with NLS-hJMY (Fig. 3c), overexpression of NLS-mJMY resulted in reduced DNA damage during the DDR (Fig. S6a–c). Interestingly, removal of JMY’s entire WH2-domain containing WCA region (NLS-ΔWCA) or ability to mediate Arp2/3-dependent actin nucleation (NLS-W981A) led to increased DNA damage in comparison to wild-type expressing cells (Fig. 5c). This suggested that JMY’s Arp2/3-dependent actin nucleation activity is involved in its ability to influence DNA repair. To explore this further we inhibited Arp2/3 activity (CK666 [24]) in U2OS cells overexpressing NLS-hJMY. Under normal growth conditions Arp2/3 inhibition had no significant impact on DNA damage, while under etoposide treatment, inhibition of Arp2/3 activity prevented the decreased DNA damage seen in the presence of NLS-JMY (Fig. 5d). In addition, JMY’s Arp2/3-dependent actin nucleation activity impacted on the expression of p53 target genes involved DNA repair (Fig. 5e). Thus, JMY-mediated Arp2/3-driven actin nucleation plays a role in DNA repair and this, in part, is mediated through its ability to influence p53-dependent transcription.

### JMY promotes cell proliferation and survival during the DDR

**Fig. 6:**
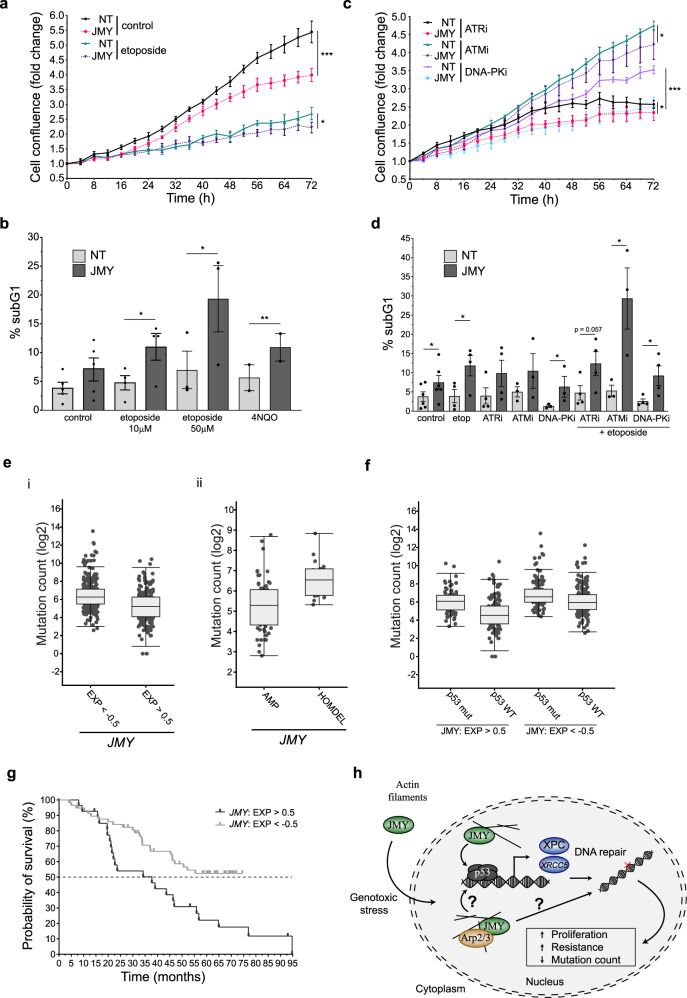
JMY enhances cell proliferation and influences cell survival during DDR. Cell confluence of U2OS cells transfected with JMY siRNA or non-targeting control and treated with either vehicle (control), etoposide (10 µM) (**a**) and ATM (ATMi; KU60019 5 µM), ATR (ATRi; AZD6738 5 µM) or DNA-PK (DNA-PKi; M3814 5 µM) inhibitors (**c**) as indicated. Graphs represent cell confluence as fold change after normalising to time zero images (mean ± SD), *n* = 3 independent experiments, representative experiment shown. **b**, **d** U2OS cells were transfected as in **a** and treated with vehicle (control), etoposide (as noted) or 4NQO (500 nM) (**c**), or ATM (ATMi, 10 μM), ATR (ATRi; 10 μM) and DNA-PK (DNA-PKi; 1 μM) inhibitors in the presence or absence of etoposide (10 μM) (**d**) before collecting for flow cytometry. Graphs represent percentage subG1 (mean ± s.e.m.), *n* = 3–5 independent experiments. **e**, **f** Median mutation count of tumours stratified by low (EXP < −0.5) versus high (EXP > 0.5) *JMY* mRNA expression levels (i), *JMY* amplification versus deletion (ii) or stratified by p53 (*TP53*) mutation status (**f**), obtained from TCGA pan-cancer dataset. **g** Kaplan–Meier survival curves for patients whose tumours express low (light grey) or high (dark grey) levels of *JMY* mRNA, obtained from TCGA pan-cancer study. **h** During DNA damage, JMY is required for the p53-mediated expression of DNA repair targets, and via its Arp2/3-dependent actin nucleation, impacts on the accumulation of DNA lesions and overall cell survival. *p < 0.05, **p < 0.01 and ***p < 0.001, Student’s *t*-test.

Given that JMY can enhance DNA repair via the p53-response, we explored the impact of JMY on cell fate during the DDR. Short-term JMY depletion had a marked effect on cell proliferation during unperturbed growth conditions with a modest but significant effect under etoposide treatment (Fig. 6a), while JMY knockout cells displayed a more dramatic decrease in proliferation under etoposide treatment (Fig. S7a). This decrease in cell proliferation was reflected in increased cell death during the DDR in both the knockdown and knockout models (Fig. 6b; Fig. S7b, e). Because JMY levels correlate with reduced DDR signalling and increased DNA damage, we reasoned that JMY may influence sensitivity to DDR inhibition. Indeed, under normal growth conditions, JMY deficiency resulted in increased sensitivity to inhibition of ATM, ATR or DNA-PK (Fig. 6c; Fig. S7c), which was further exacerbated during the DDR (Fig. 6d; Fig. S7d).

In patient samples, we found that across all cancer types (TCGA pan cancer), tumours with lower *JMY* mRNA expression or homozygous deletion contained an increased mutation count (Fig. 6ei, ii). Moreover, further stratification based on *p53 (TP53)* mutation status revealed that those tumours with higher *JMY* mRNA levels along with wild-type *p53* expression exhibited a significantly lower mutation count (Fig. 6f). Given that JMY reduced DNA damage accumulation, increased resistance to chemotherapeutic drugs, and reduced overall mutation count, we hypothesised that tumours with higher *JMY* expression might exhibit a more aggressive phenotype resulting in poorer patient survival. Indeed, patients with higher *JMY* mRNA expression presented substantially lower overall survival (Fig. 6g). Thus, our results suggest that JMY can enhance cell survival during the DNA damage response through impacting on p53-mediated gene expression and DNA repair and this is reflected in patient outcomes in human cancers.

## Discussion

The tumour suppressor p53 plays a key role in DNA repair both indirectly by triggering cell cycle arrest as well as directly via transcriptional activation of DNA repair genes [3, 13]. Here we present a novel role for JMY in DNA repair during the DDR and show how the lack of JMY compromises the expression of p53 targets involved in DNA repair and hinders DDR signalling leading to the accumulation of DNA damage. Moreover JMY’s Arp2/3-dependent actin nucleation activity enhances DNA repair and p53 target gene expression. Ultimately depletion of JMY sensitises cells to DDR inhibitors and impacts on cell survival and this is reflected in human cancers where a lack of, or decreased, *JMY* mRNA expression results in better overall survival and increased tumour mutation count (Fig. 6h).

JMY is an actin nucleator and DNA damage-responsive protein that undergoes nuclear accumulation upon genotoxic stress [14, 17, 25]. JMY supports both Arp2/3-dependent and independent actin nucleation [15] and in the cytoplasm JMY can enhance autophagy to promote cell survival [16]. In the nucleus, JMY influences p53 transcriptional activity where previous studies demonstrated JMY could enhance p53-driven expression of *Bax* [17]. Our transcriptomic analysis supports a wider role for JMY in transcriptional regulation and ability to influence p53-dependent gene expression, in particular genes involved in DNA repair. A multitude of factors regulate target gene selection by p53, including p53 activation and stability which can be influenced by post-translational modifications, p53 cofactors and binding proteins [2]. p53 is regulated by all three DDR kinases (ATM, ATR and DNA-PK) resulting in stabilisation and enhanced nuclear localisation [2]. JMY’s impact on DDR signalling influences p53 recruitment to target promoters (Fig. 2e). Further, JMY’s impacts on p53-dependent gene expression will also influence downstream DDR signalling as well as DNA repair. *XRCC5* (Ku80), for example, together with XRCC6 (Ku70) forms the DNA-binding Ku heterodimeric complex that forms a scaffold to recruit other repair proteins to DNA damage sites, including the DDR kinase DNA-PK [26]. A reduction in DNA-PK recruitment to damaged chromatin in the absence of JMY could explain the sensitivity of JMY knockout cells to DNA-PK inhibition (Fig. S7d). Overall, we have expanded our understanding of the role of JMY during the DDR to show that JMY reduces DNA damage accumulation by both positively impacting on p53-dependent transcriptional activity and DDR signalling.

Although incompletely understood, we know that different stressors can influence the subcellular localisation of JMY. For example, certain genotoxic stressors result in JMY nuclear accumulation (Fig. 1a; Fig. S2a; [25]), while metabolic stressors such as starvation lead to JMY association with cytoplasmic autophagosomes [16]. Recently, JMY was shown to influence p53-dependent apoptosis through cytoplasmic Arp2/3-dependent actin nucleation and influence several steps in the mitochondrial-dependent apoptotic process [27], thus adding further complexity to JMY’s cytoplasmic role as well as its overall impact on cell fate. It is clear that JMY can influence cell survival via a number of different means and it is likely that differences in the type of stressor and duration and dose will impact on JMY’s subcellular localisation and thus activity in the cell. Studies have shown that p53 activity and outcome is influenced by the duration and type of stressor. For example, pulsating p53 levels have been shown to activate a transient expression of DNA repair and cell cycle arrest genes [28, 29], while more sustained expression of p53, leads to the activation of pro-apoptotic genes [29]. Thus it is likely that for a shift between DNA repair to apoptotic p53-dependent gene expression, the levels of p53 must exceed a time-dependent threshold [29, 30]. Further studies are required to refine our understanding of how temporal and dose-dependent effects influence JMY-mediated impacts on p53 activity during the DNA damage response and how this modulates gene expression programmes to influence outcome. Moreover, JMY promotes cell survival through its influence on nuclear activities such as transcriptional regulation as well as through its positive impact on autophagy [16]. It will be relevant for future studies to assess the impact of JMY on autophagy during the DDR as, for example, etoposide can enhance autophagy [31] and our transcriptomic analysis also identifies autophagy as a significantly altered pathway during the DDR (Fig. 1c).

Our data support a role for JMY’s actin nucleation activity in both DNA damage accumulation and transcriptional regulation. Previous work showed that JMY’s ability to enhance p53-dependent activity in *Bax*-luciferase reporter assays was hindered with latrunclin A treatment (to prevent all cellular actin nucleation) but JMY’s Arp2/3-dependent nucleation activity had no effect [14]. It is likely that JMY’s activity at target genes will be promoter-specific and, for example, JMY may result in differential recruitment of p53 or actin to DNA repair versus apoptotic targets. Future studies are needed to explore the impact of nuclear JMY on actin recruitment to target genes and how this effects gene expression and cell survival during stress. Importantly, a growing body of evidence implicates WASp family proteins in different aspects of DNA repair. For example, in lymphocytes WASp deficiency promotes R loop accumulation leading to DNA damage via its impact on transcription [32]. Interestingly, nuclear WASp has also been shown to facilitate homology-directed repair via Arp2/3-dependent F-actin formation to influence break clustering of double-strand breaks [19]. More recently nuclear WASH (a member of the WASp family of actin nucleators) was shown to promote DNA repair directly at double-strand breaks through interaction with components of the non-homologous end-joining machinery mediated by its Arp2/3-dependent actin nucleation ability [33]. Overall, data supports the fact that actin polymerisation and the recruitment of actin nucleators directly to DNA lesions are crucial for the correct repair of DNA breaks [18]. Whether JMY also plays a role in DNA repair via direct recruitment to DNA lesions requires further studies to provide an improved mechanistic understanding of JMY’s nuclear function. Nonetheless, our work adds to the evidence that actin nucleation mediated by nuclear proteins plays key roles in cellular outcome during the DNA damage response.

Together, we demonstrate a wider role for JMY in p53-dependent gene expression, and through Arp2/3-dependent actin nucleation, JMY impacts on the accumulation of DNA damage and overall cell survival (Fig. 6h). Tumour cells commonly present defects or reduced expression in DNA repair genes [11], leading to dependency on compensatory and often less efficient DNA repair and survival pathways that can be exploited in the clinic. Our study provides a link between JMY and nuclear actin dynamics in DNA repair during the p53-mediated DDR. This provides further insights into regulation of p53 activity in human cancer which could ultimately lead to clinical opportunities to manipulate p53 and DDR pathways to maximise patient benefit.

## Materials and Methods

### Plasmids, antibodies and reagents

The following plasmids have been previously described: pcDNA3 HA-mJMY, HA-NLS-mJMY, HA-NLS-ΔWH2, and HA-NLS-W981A [14]. FLAG-NLS-hJMY and HA-hJMY were created by sub-cloning human JMY (ORF clone NM_152405, Origene) into FNpcDNA3 (Gift from Robert Oshima, Addgene plasmid #45346) or pCEFL-HA (Gift from Eric O’Neill, University of Oxford) and verified by sequencing. A complete list of antibodies is detailed in SI Table 3. Etoposide, ATM (KU60019), ATR (AZD6738) and DNA-PK (M3814) inhibitors (Cambridge biosciences) and 4NQO (4-Nitroquinoline N-oxide, Sigma-Aldrich) were used at different concentrations as detailed in the figure legends.

### Cell lines and generation of stable cell lines

U2OS, MCF7 (Public Health England), Saos2 (Gift from Glen Kirkham, Nottingham Trent University), HAP1 parental and HAP1 JMY knockout (KO) cells were grown in DMEM (4.5 g/L glucose with glutamax), supplemented with 5% foetal bovine serum without antibiotics under 5% CO_2_. Chronic myelogenous leukaemia HAP1 parental and JMY KO cells were purchased from Horizon Discovery (Product ID: HZGHC002630c002). HAP1 JMY KO cells were obtained by CRISPR/Cas9 gene editing using sgRNA: AGTGCGGGCCAAACCCATCC generating a 10 bp deletion in the first coding exon of *JMY*. Stable U2OS cells expressing JMY constructs were obtained after transfecting cells with the appropriate construct and selecting with G418 at 500 µg/mL.

### Transfection

Plasmid and siRNA transfections were performed using TransIT-X2 (Mirus) and Optimem (ThermoFisher). Plasmid transfections were performed using 200 ng plasmid and siRNA transfections were carried out using 25 nM siRNA. Human JMY siRNA has been previously described [34] and siRNA AllStars  (5′-UUCUCCGAACGUGUCACGU(UU)-3′) was used as control non-targeting siRNA.

### Comet assays and quantification

Single-cell alkaline comet assays were performed following the manufacturer’s instructions (R&D Systems). Coverslips were stained with 2 µg/mL Hoechst-33342 for 45 minutes at room temperature before imaging. Images were obtained using a Leica DMi8 inverted fluorescence microscope with 20x or 40x dry lenses. A minimum of 50 comets were quantified per condition. Comet tails were quantified using the OpenComet plugin [35] from ImageJ/Fiji [36].

### RNA isolation, reverse transcription and RT-qPCR

RNA was isolated using TRIZol reagent (Sigma-Aldrich), followed by chloroform extraction, precipitation with isopropanol, 70% ethanol wash before RNA was resuspended in water. 1 µg of RNA was reverse transcribed using random hexamers and MMLV-RT (New England Biolabs). RT-qPCR was performed using Brilliant III Ultra-Fast SYBR qPCR (Agilent Technologies) and quantified using the 2^−^^ΔΔCt^ method [37]. GAPDH was used as an internal control. Primer sequences are detailed in SI Table 4.

### Immunoblotting and quantification

Cells were seeded into 6 cm dishes and treated as appropriate before harvesting in TNN buffer (150 mM NaCl, 50 mM Tris-HCl pH 7.4, 5 mM EDTA, 0.5% NP40, 50 mM NaF, 0.2 mM Na_3_VO_4_, in the presence of protease inhibitors). Protein quantification was performed using Bradford Reagent (Sigma-Aldrich) and lysates were run using SDS-PAGE, transferred to nitrocellulose membranes and probed overnight at 4 °C with primary antibodies diluted in 5% skimmed milk in 0.1% Tween-20 in PBS (v/v). Membranes were washed extensively and probed with secondary antibodies before detection by enhanced chemiluminescence (ECL) using ChemiDocTM XRS+ with Image LabTM software (Bio-Rad). Band quantification was performed using Fiji/ImageJ [36]. Uncropped blots are presented in Fig. S8.

### Immunostaining and quantification

Cells were seeded onto 13 mm glass coverslips, fixed in 3.7% formaldehyde for 10 minutes and permeabilised with 0.5% Triton X-100 in phosphate-buffered saline (PBS) (v/v) for 5 minutes at room temperature. Coverslips were incubated overnight at 4 °C with primary antibodies, extensively washed with 0.025% Tween-20 in PBS (v/v) and further incubated with secondary antibodies at room temperature for 30 minutes. Coverslips were washed and mounted on microscope slides using Vectashield with DAPI (4,6-diamino-2-phenylindole) for nuclei visualisation. Images were obtained using a Leica DMi8 inverted fluorescence microscope. Images were quantified using ImageJ/Fiji [36] unless otherwise specified. A minimum of 100 cells were quantified per condition.

Foci were quantified using the FindFoci plugin [38] from ImageJ/Fiji [36]. Briefly, two separate folders containing the nuclei (stained with DAPI) and the foci images (antibodies specified in the figure legends) were used as input files. Masks of the nuclei were obtained using the auto-threshold otsu_4_level to distinguish between the nuclei signal and the background. Clumped nuclei were separated using the watershed function from ImageJ/Fiji. Foci and fluorescence signal were quantified and sorted using the minimum_above_saddle and average intensity minus background functions, respectively. Fluorescence quantification was obtained after normalising with the number of cells per field using the nuclei pictures as input, and the analyse particles function from ImageJ/Fiji. Results were exported to Excel and graphs and statistical analysis were conducted using GraphPad Prism 9.0.2.

Nuclear accumulation of JMY was measured using a modified version of the ‘Human C-N translocation’ CellProfiler pipeline [39]. Briefly, two separate folders containing the nuclei (stained with DAPI) and HA-hJMY signal, detected with a mouse anti-HA antibody, were used as input files. Masks of the nuclei were obtained using a global three-class Otsu threshold method to distinguish between the nuclei signal and the background. Clumped nuclei were separated using a shape-smoothing function and nuclei at the image borders were discarded. JMY signal was measured using the same CellProfiler module and threshold using the nuclei masking (generated in the previous step) to quantify the nuclear versus cytoplasmic signal. Results were exported to Excel and graphs and statistical analysis were conducted using GraphPad Prism 9.0.2.

### Cell proliferation assays

Cells were seeded into 96-well plates at 5000 cells per well one day prior to performing cell proliferation assays using the IncuCyte S3 live-cell analysis system. Cells were treated with either vehicle control or specific drug treatments (as noted) and were imaged every 4 h for 72 h. Four images were taken per well, and treatments were performed in quadruplicate. Quantification was performed by masking the phase contrast cell confluence after normalising against time zero images (set at 1 for time zero) for each treatment set using the Incucyte live-cell analysis system. Masks were obtained from 16 images per time point and treatment.

### Flow cytometry

Cells were seeded into 6 cm dishes and treated as appropriate for 30 h before harvesting. Growth media was collected and combined with adherent cells, pelleted at 500 x *g* for 3 minutes at 4 °C, washed once with cold PBS and fixed in ice-cold 70% ethanol in PBS (v/v) overnight at 4 °C. Fixed cells were washed and stained with 2% (v/v) propidium iodide, including 125 U/mL DNAse-free RNAse A. Analysis was performed using flow cytometry (Accuri C6, BD Bioscience).

For monitoring apoptosis, HAP1 parental and HAP1 JMY knockout cells were seeded into 6 cm dishes at a concentration of 2.5 × 10^5^ cells per dish 48 h before treatment. Cells were treated as appropriate for 30 h before harvesting. Growth media was collected and combined with adherent cells, pelleted at 500 x *g* for 3 minutes at 4 °C and washed once with 1x annexin-V binding buffer (10 mM HEPES, 140 mM NaCl and 2.5 mM CaCl_2_). Cells were resuspended in 1x annexin-V binding buffer to a final concentration of 10^6^ cells/mL, and 100μL of cells were stained with annexin-V conjugated with FITC (Invitrogen) and 1μg/mL of propidium iodide in the presence of DNAse-free RNAse A for 30 minutes at room temperature. Analysis was performed using flow cytometry (Accuri C6, BD Bioscience).

### Chromatin immunoprecipitation (ChIP)

HAP1 cells were seeded into 10 cm dishes and treated with etoposide (500 nM) for 6 h. Cells were cross-linked with 1% formaldehyde for 10 minutes before quenching with 0.125 M glycine. Pellets were permeabilised in lysis buffer I (5 mM Tris-HCl pH 8, 85 mM KCl and 0.5% NP40, in the presence of protease inhibitors) to obtain nuclei which were subsequently lysed using nuclei lysis buffer (50 mM Tris-HCl pH 8.1, 10 mM EDTA and 1% SDS, in the presence of protease inhibitors). Samples were sonicated using Bioruptor® Pico (Diagenode) for 10–20 cycles (30 s on, 30 s off). Chromatin samples were diluted 1:5 with IP dilution buffer (0.01% SDS, 1% Triton X-100, 1.2 mM EDTA, 16.7 mM Tris-HCl pH 8.1, 167 mM NaCl) prior to immunoprecipitation using 30 μL protein A/G slurry beads in the presence of 2μg of anti-p53 or mouse non-specific IgG antibody (SI Table 3). Samples were washed 4X each with low salt buffer (20 mM Tris-HCl pH 8.1, 150 mM NaCl, 2 mM EDTA, 0.1% SDS and 1% Triton X-100) and LiCl buffer (0.25 M LiCl, 1% NP40, 1% Na-deoxycholate, 1 mM EDTA and 10 mM Tris pH 8.1), followed by 2X washes with TE buffer before reverse cross-linking and RNAse digestion at 55 °C for 3 h followed by overnight at 65 °C. DNA was isolated with Qiaquick PCR purification kit (Qiagen) according to manufacturer’s instructions. ChIP samples were analysed by qPCR using Brilliant III Ultra-Fast SYBR (Agilent). Primer sequences are detailed in SI Table 4.

### RNA-sequencing and bioinformatic analysis

U2OS cells were seeded into 10 cm dishes, transfected with 12.5 nM JMY or non-targeting siRNA for 72 h and treated with 50 µM etoposide for the last 6 h before harvesting and storing pellet at −80 ^o^C (*n* = 3 independent biological repeats). RNA was isolated using the ReliaPrep kit (Promega) following the manufacturer’s instructions. 3 µg of RNA were used for building the library according to NEBNext® Ultra™ RNA Library Prep Kit (New England Biolabs) following the manufacturer’s recommendations. After cluster generation, libraries were sequenced on a HiSeq platform (Illumina), generating 30 million paired-end reads of 150 bp length, using the services of Novogene Co., Ltd. The quality of the reads and the removal of adaptor sequences was performed using FastQC (GALAXY Version 0.72). Clean reads were mapped to the reference human genome (hg38) using TopHat2 (GALAXY Version 2.1.1) [40], and the quality of the mapping was analysed using QualiMap RNA-Seq QC (GALAXY Version 2.2.2d). BAM files were sorted by coordinates using SortSam (GALAXY Version 2.18.2.1) and quantified using HTseq-count (GALAXY Version 0.9.1) [41] using the reference transcriptome (v82). DESeq2 (GALAXY Version 1.1.0) [42] was used to normalise and calculate the differential transcript expression between JMY and non-targeting siRNA cells. A final list of differentially expressed genes was obtained using q-value < 0.001.

Pathway enrichment analysis was performed following Reimand and colleagues’ protocol [43]. Briefly, differentially expressed genes were used as input for the g:GO analysis from gProfiler [44] with a significant threshold of Benjamini-Hochberg FDR < 0.05. Enriched pathways and gene ontologies were obtained from the KEGG, REACTOME and the Gene Ontology Consortium databases, respectively.

To explore the role of JMY in patient outcomes in human cancers, we used the cBioPortal for Cancer Genomics database (http://www.cbioportal.org; [45]). Data was obtained using the ICGC/TCGA pan-cancer cohort [46], including 2,922 samples from 2583 patients. Samples were manually grouped based on (i) *JMY* expression levels (mRNA expression z-scores, high: EXP > 0.5 or low: EXP < −0.5) or *JMY* copy number (amplification: AMP or homozygous deletion: HOMDEL). Groups were further split based on p53 mutation status (wild-type: WT or mutant: mut) using cBioportal Onco Query Language. Clinical data were retrieved, including Kaplan–Meier patient survival curves and mutational counts.

### Statistical analysis

Statistical analysis was performed using GraphPad Prism 9.0.2. All data were tested for normal distribution. At least three independent experiments were performed, and individual data points are represented. Results with error bars represent mean ± standard error of the mean (s.e.m.), unless otherwise specified in the figure legends. Statistical analyses were performed using at least 3 independent biological repeats (n values). For quantification of fluorescence in Figs. 1a, 4aiii, 4biii, S2a, cell numbers (N values) were used for statistical analyses by pooling the data from at least 3 independent biological repeats with a minimum of 100 cells/images per condition for each repeat. The differences between two groups were analysed by unpaired two-tailed Student’s t-test for normalised data, and Mann–Whitney U test for non-normalised data. All values were considered significant with *p*-value < 0.05.

## Supplementary information

Figure S1

Figure S2

Figure S3

Figure S4

Figure S5

Figure S6

Figure S7

Original Data Files

SI Table 1

SI Table 2

SI Table 3

SI Table 4

SI Figure legends no mark up

CDD checklist

## Data Availability

RNA-sequencing data have been deposited to Array Express under accession number 513 E-MTAB-12059. Constructs generated in this study are available upon request.
