# Supplementary figures and images for: p53-dependent DNA repair during the DNA damage response requires actin nucleation by JMY
Source: Cell Death Differ. 2023 May 4;30(7):1636–47. doi: 10.1038/s41418-023-01170-9 (PMC10307838; doi:10.1038/s41418-023-01170-9)

SI Figure 1

a

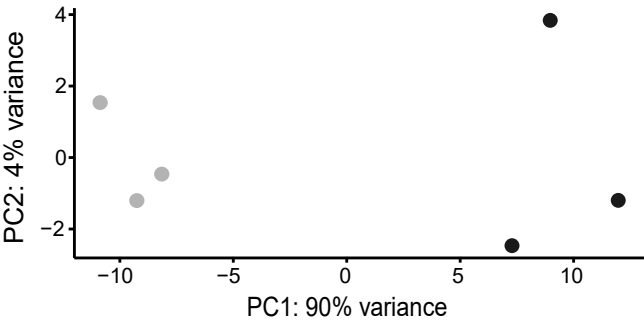

b

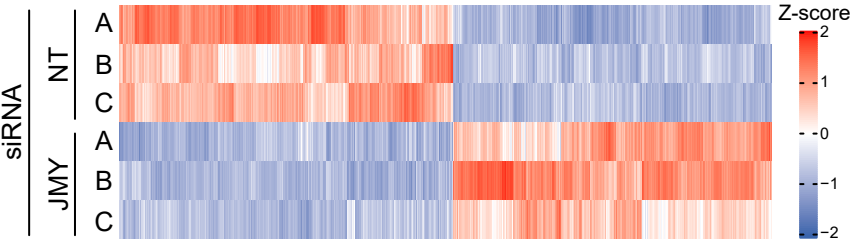

c

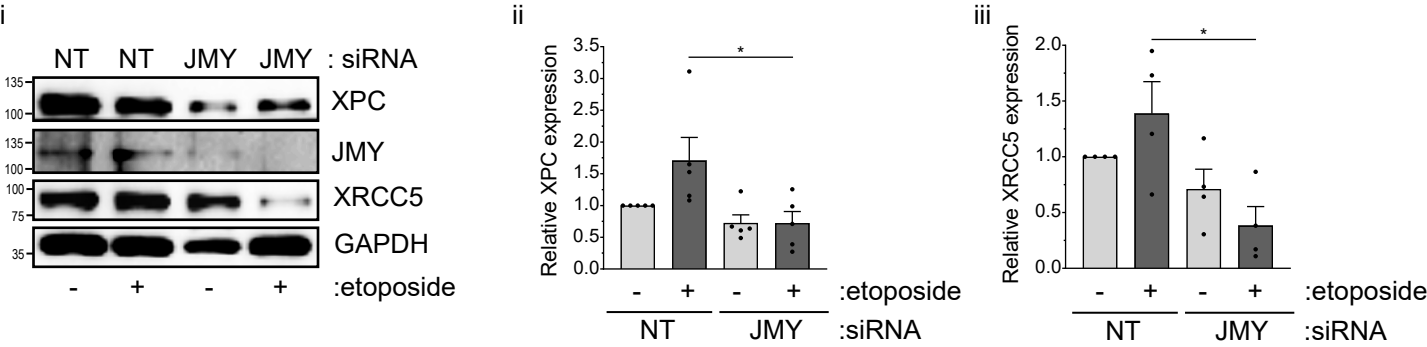

Supplement: Supplementary file 1 — Figure S1 [file 41418_2023_1170_MOESM1_ESM.pdf]

SI Figure 2

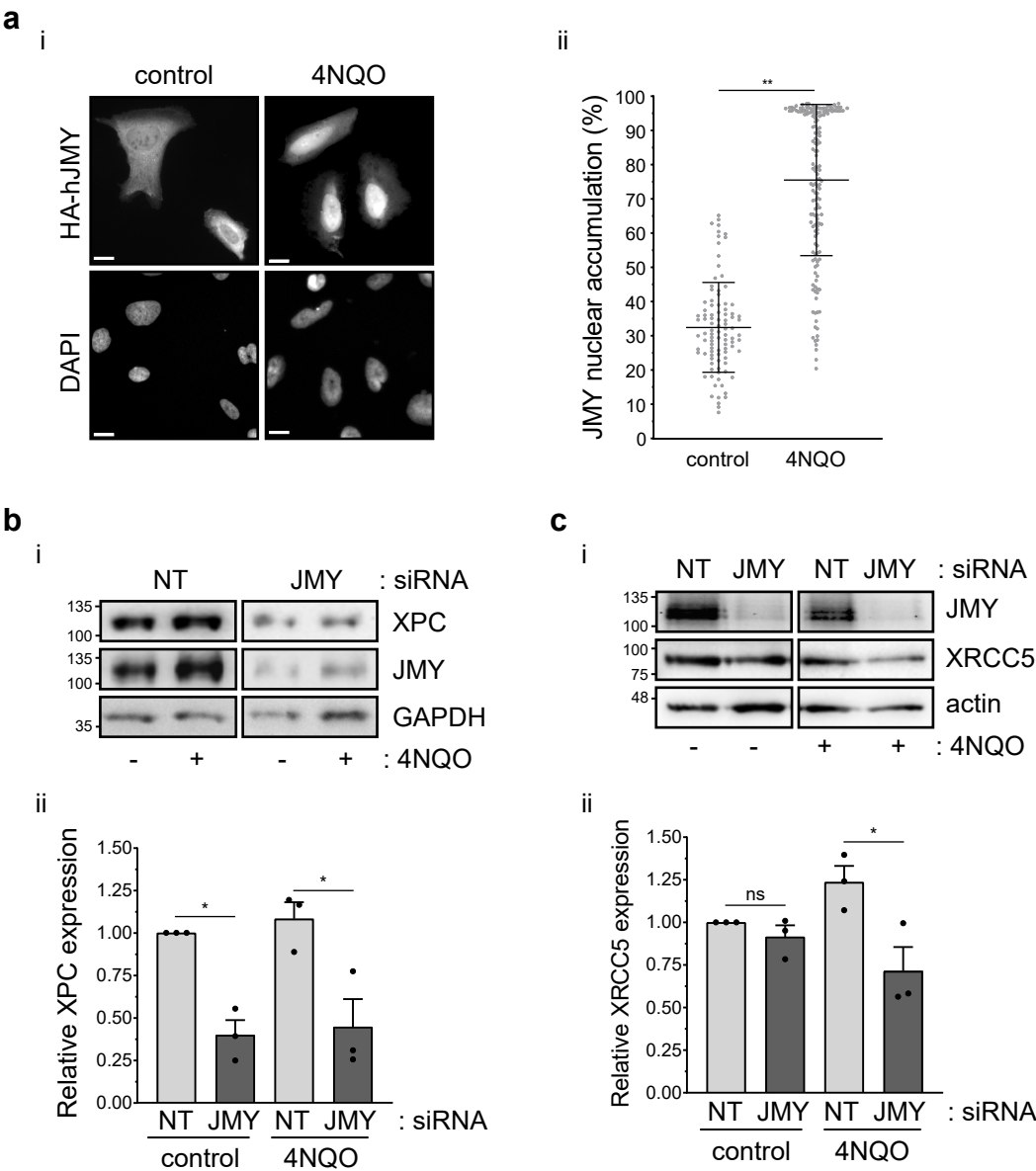

Supplement: Supplementary file 2 — Figure S2 [file 41418_2023_1170_MOESM2_ESM.pdf]

SI Figure 3

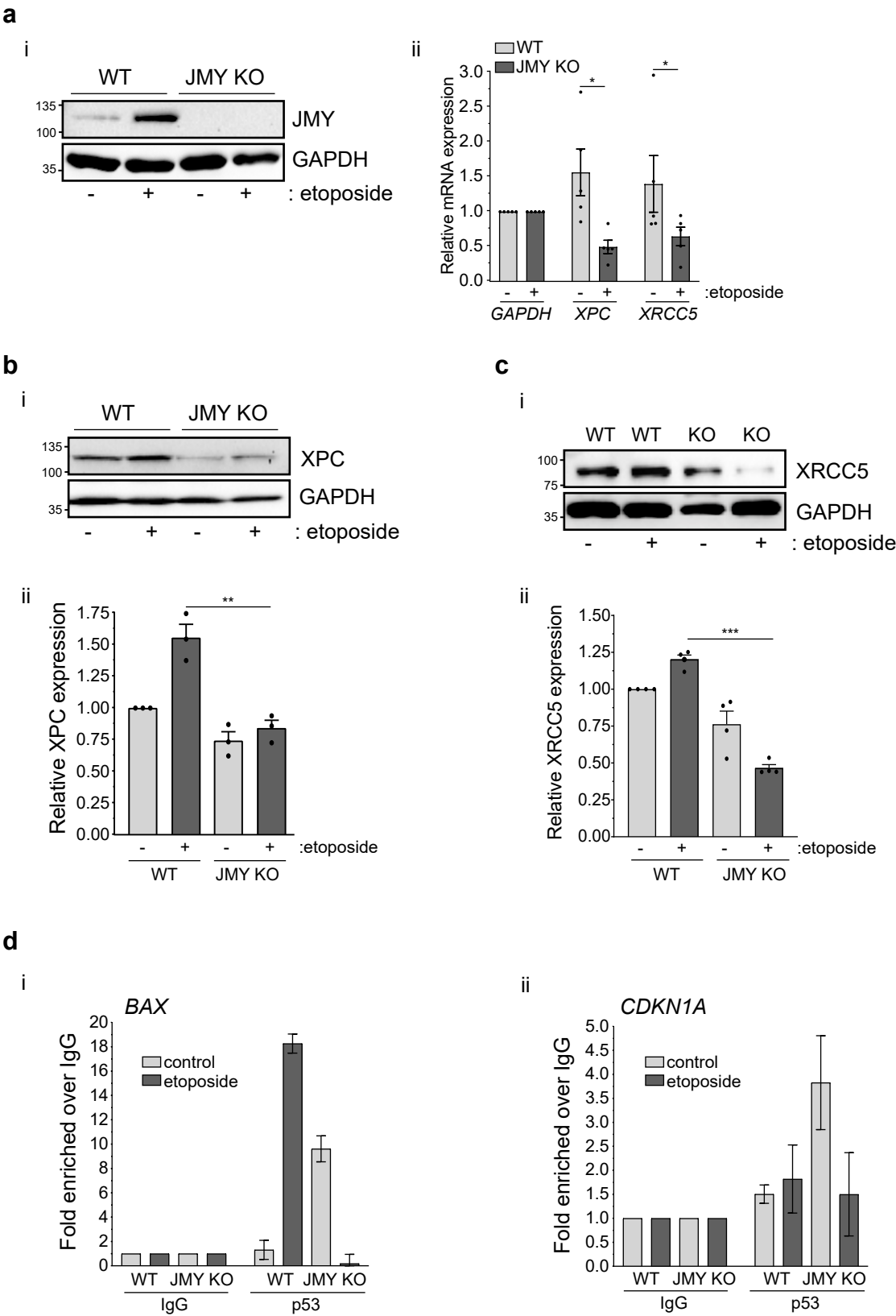

Supplement: Supplementary file 3 — Figure S3 [file 41418_2023_1170_MOESM3_ESM.pdf]

SI Figure 4

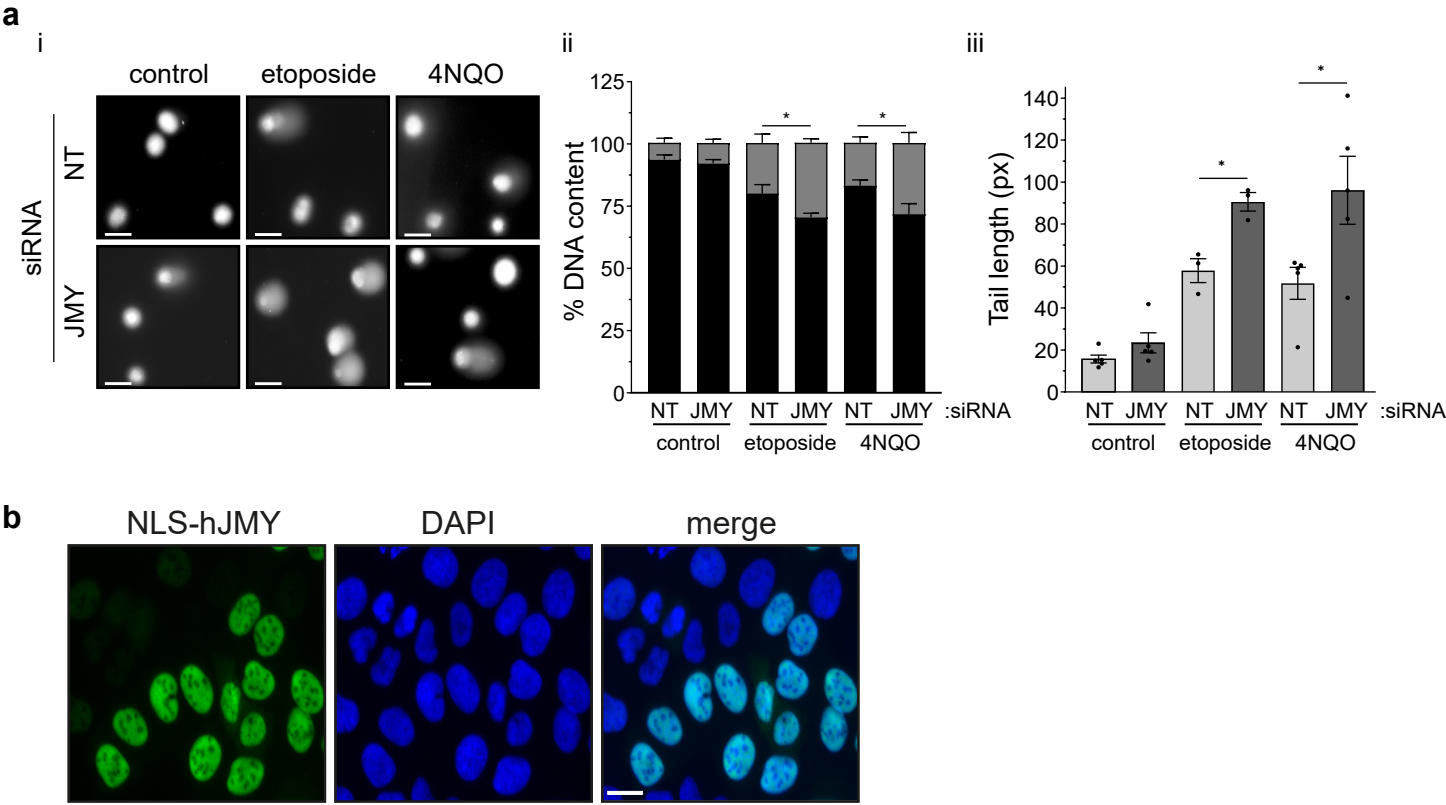

Supplement: Supplementary file 4 — Figure S4 [file 41418_2023_1170_MOESM4_ESM.pdf]

SI Figure 5

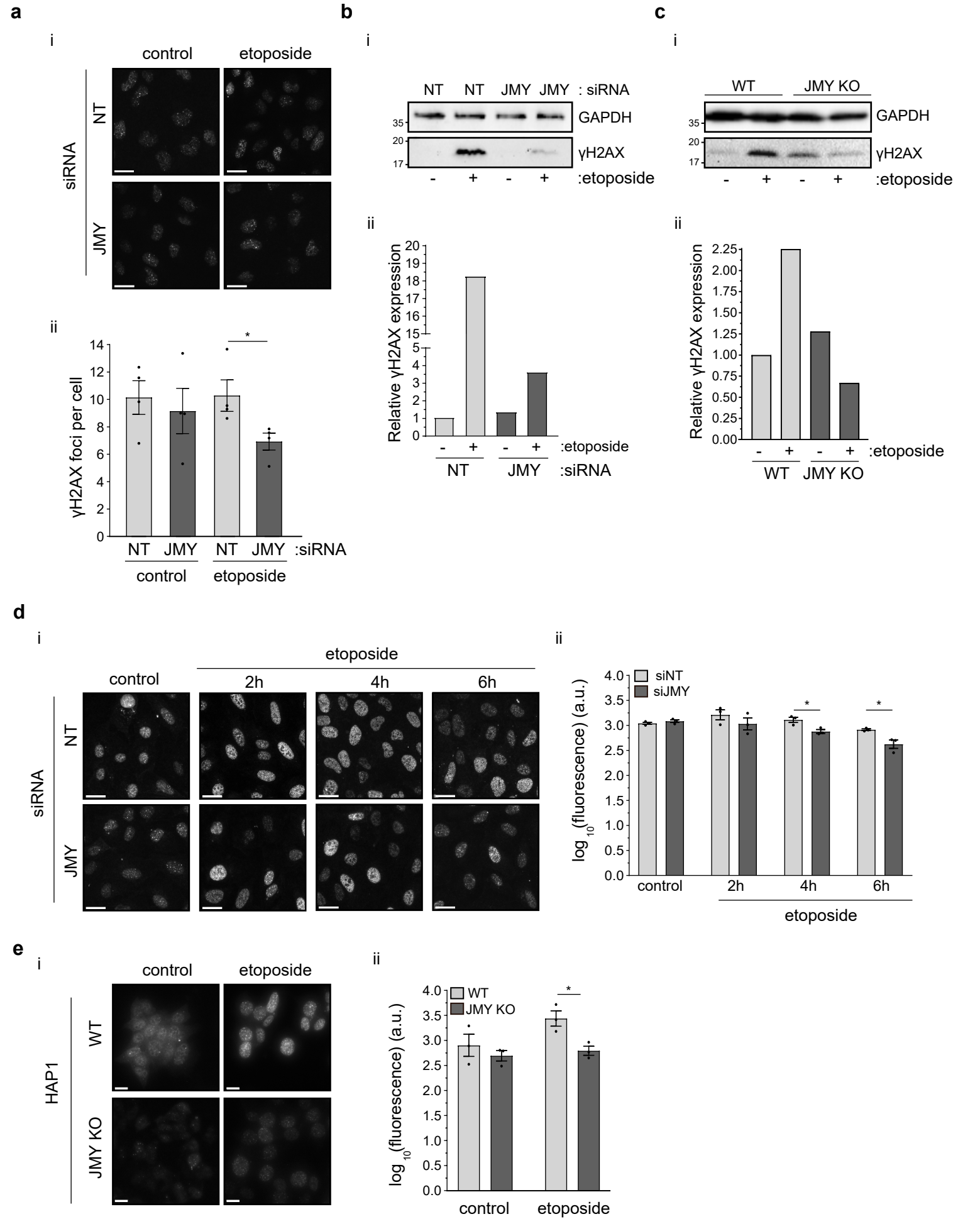

Supplement: Supplementary file 5 — Figure S5 [file 41418_2023_1170_MOESM5_ESM.pdf]

SI Figure 6

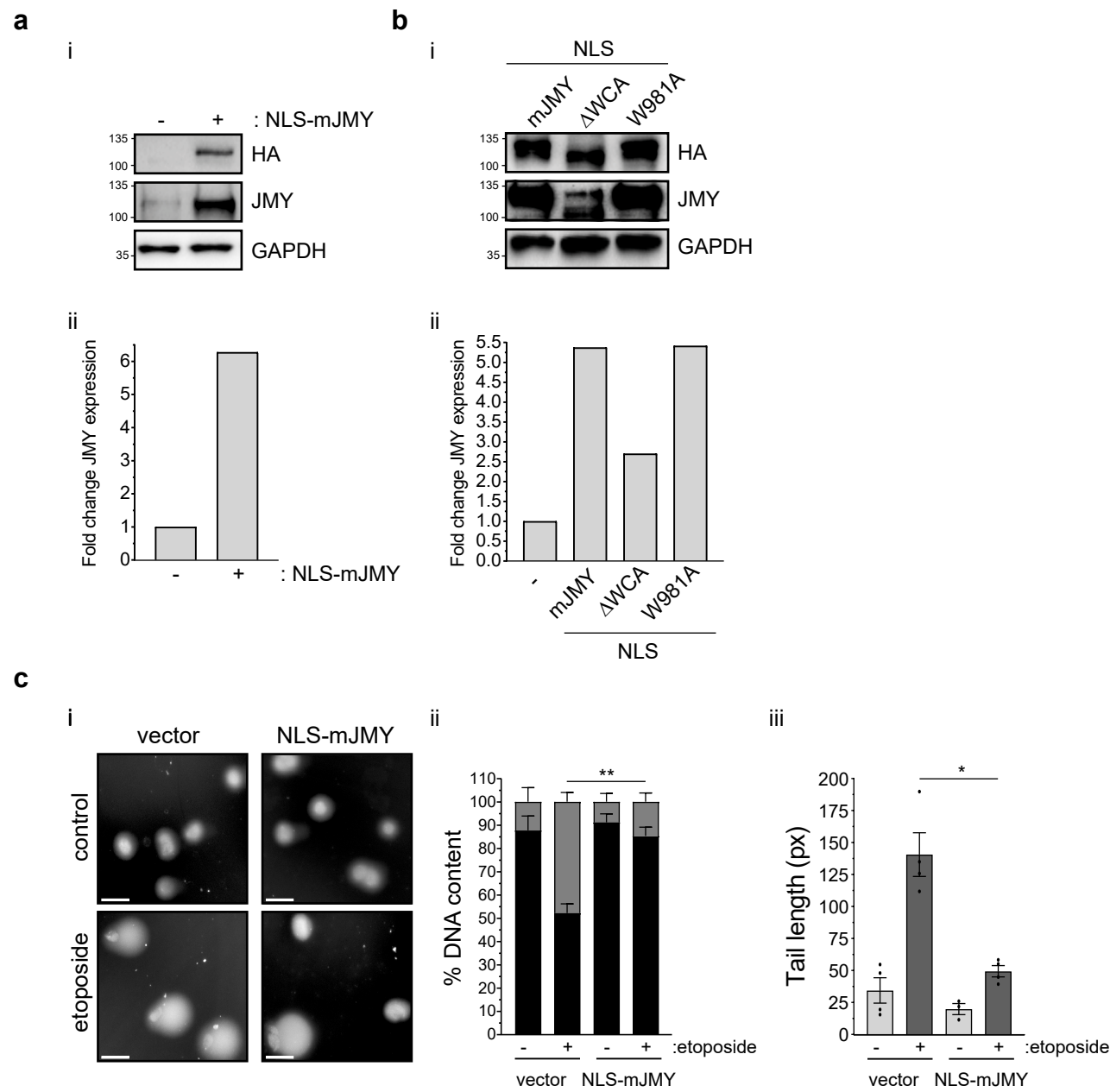

Supplement: Supplementary file 6 — Figure S6 [file 41418_2023_1170_MOESM6_ESM.pdf]

SI Figure 7

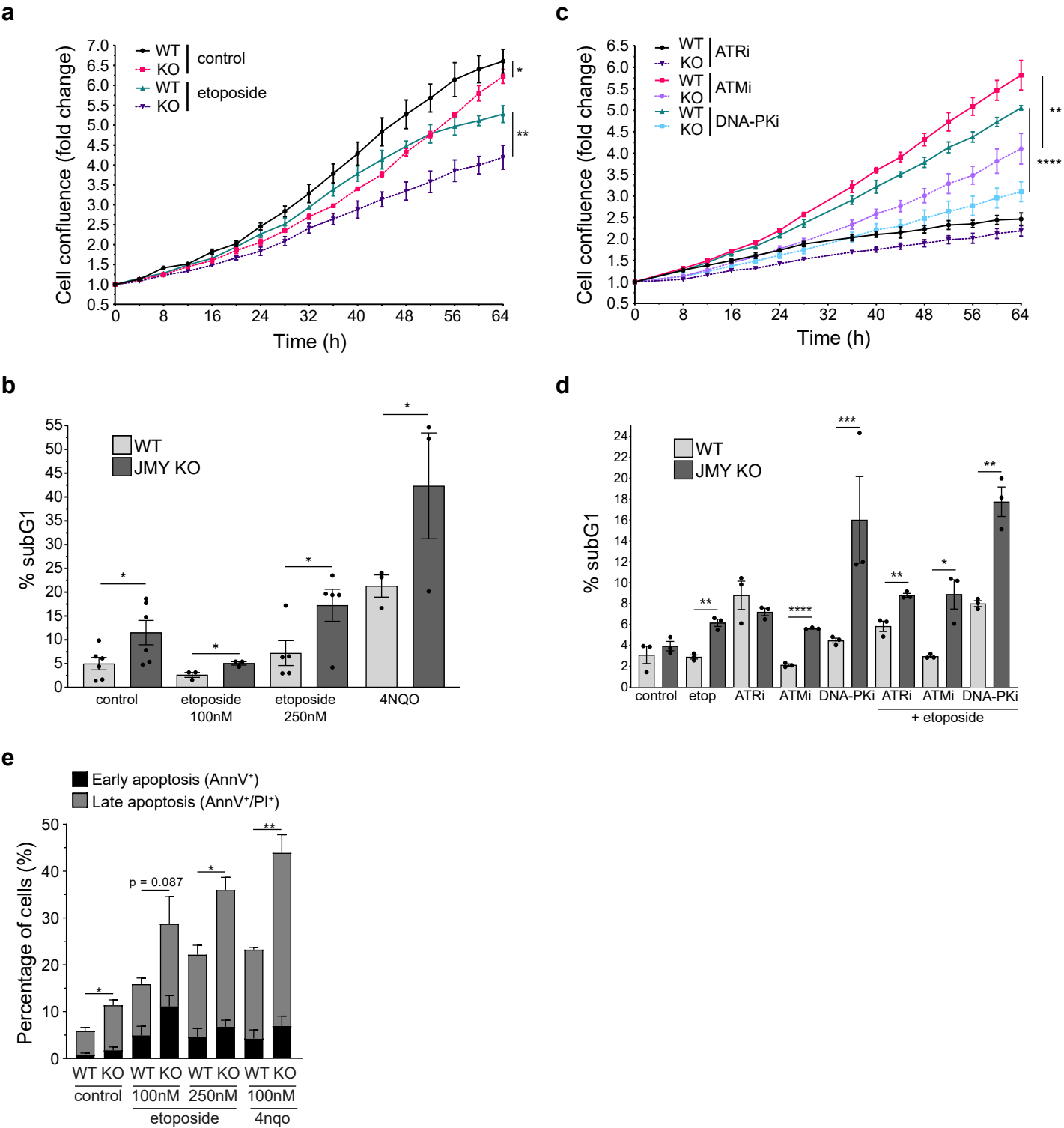

Supplement: Supplementary file 7 — Figure S7 [file 41418_2023_1170_MOESM7_ESM.pdf]
