# Supplementary material for: p53-dependent DNA repair during the DNA damage response requires actin nucleation by JMY
Source: Cell Death Differ. 2023 May 4;30(7):1636–47. doi: 10.1038/s41418-023-01170-9 (PMC10307838; doi:10.1038/s41418-023-01170-9)

Figure 2a

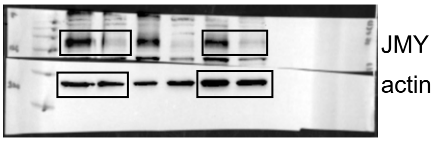

Figure 2b

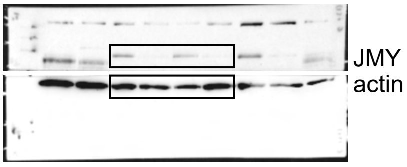

Figure 2c

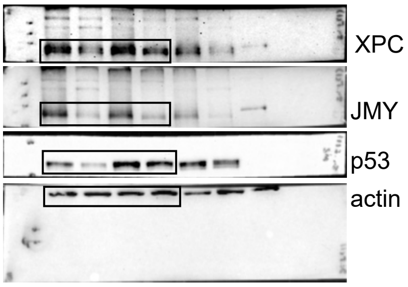

Figure 2d

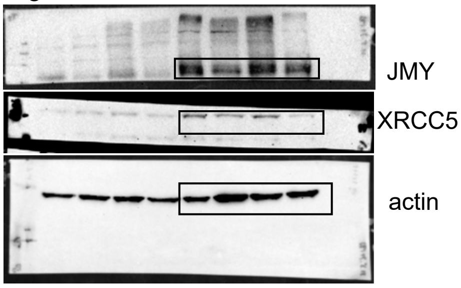

Figure S1c

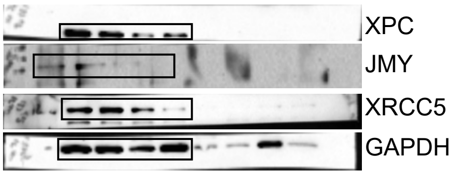

Figure S2b

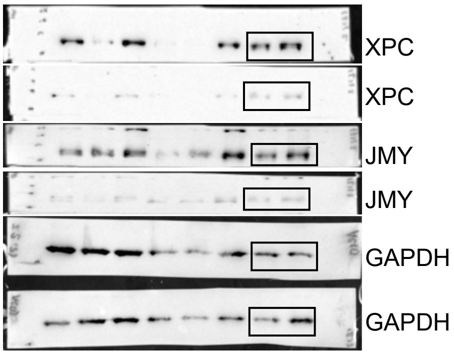

Figure S2c

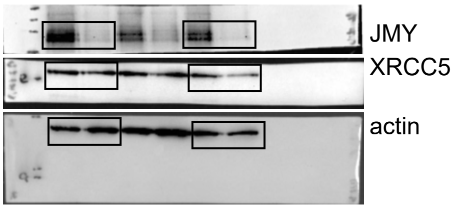

Figure S3a

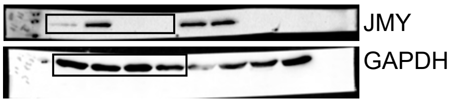

Figure S3b

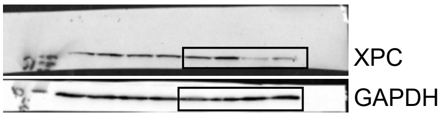

Figure S3c

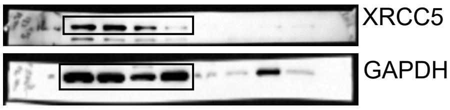

Figure S5b

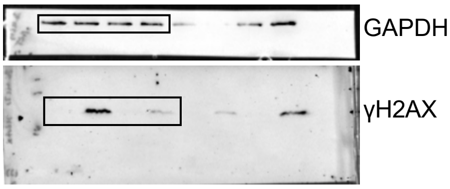

Figure S5c

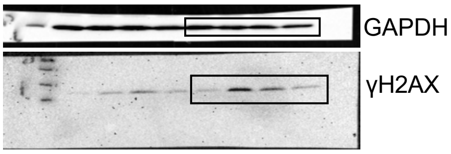

Figure S6a

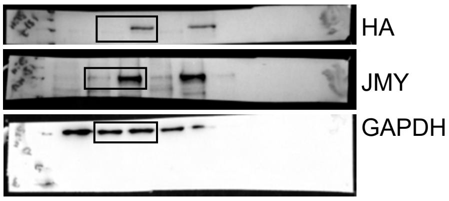

Figure S6b

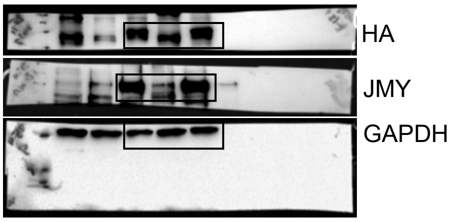

Supplement: Supplementary file 8 — Original Data Files [file 41418_2023_1170_MOESM8_ESM.pdf]
