# Supplementary material for: p53-dependent DNA repair during the DNA damage response requires actin nucleation by JMY
Source: Cell Death Differ. 2023 May 4;30(7):1636–47. doi: 10.1038/s41418-023-01170-9 (PMC10307838; doi:10.1038/s41418-023-01170-9)
Supplement: Supplementary file 13 — SI Figure legends no mark up [file 41418_2023_1170_MOESM13_ESM.docx]

**SI Figure legends**

**Figure S1. JMY enhances the expression of p53-dependent DNA repair genes. a)** Principal component analysis (PCA) of transcript expression profiles (DESeq2) from RNA-seq data. PCA shows independent clusters between U2OS cells transfected with non-targeting (light grey) or JMY siRNA (dark grey) (n=3 independent samples per condition). **b)** Transcriptomic changes influenced by JMY for upregulated (red) or downregulated (blue) targets (q-value < 0.001). **c)** MCF7 cells were transfected with JMY or non-targeting siRNA for 72h and treated with either vehicle (-, control) or etoposide (+, 50µM) for the last 6h before XPC (ii) and XRCC5 (iii) protein expression was monitored. Graph represents expression levels after normalising for GAPDH (mean ± s.e.m.). n = 4-5 independent experiments, * p < 0.05, Student’s t-test.

**Figure S2. JMY nuclear accumulation and impact on gene expression under 4NQO treatment. a)** i) U2OS cells expressing HA-tagged wild-type human JMY (HA-hJMY) were treated with vehicle (control) or 4NQO (100nM) for the last 16h. JMY was detected using anti-HA antibody. ii) Quantification of JMY nuclear versus cytoplasmic accumulation (mean ± SD). N = >150 cells per treatment. Scale bar = 10μm. ** p < 0.0001, Mann-Whitney test. **b)** and **c)** U2OS cells were transfected with JMY or non-targeting siRNA for 72h and treated with vehicle (-, control) or 4NQO (+, 100nM) for the last 16h. i) Western blot shows XPC (**b**) or XRCC5 (**c**) expression with JMY depletion. ii) Graphs represent quantification of XPC or XRCC5 levels after normalising for loading control (mean ± s.e.m.), n=3 independent replicates, * p < 0.05, Student’s t-test.

**Figure S3. JMY ablation reduces the expression of DNA repair genes. a)** HAP1 parental (WT) and JMY knockout (JMY KO) cells were treated with either vehicle (-) or etoposide (500nM) for 6h before RNA or protein extraction. i) Western blot shows JMY in parental HAP1 but not JMY knockout cells. ii) RT-qPCR results demonstrate changes in mRNA expression represented as fold mRNA expression relative to vehicle control after normalising with *GAPDH* (mean ± s.e.m.). n=5 independent experiments. **b)** and **c)** HAP1 parental (WT) and JMY knockout (JMY KO) cells were treated with vehicle (-) or etoposide (500nM) for 6h before protein extraction. i) Western blots represent XPC (**b**) and XRCC5 (**c**) levels. ii) Graphs represent expression levels after normalising with loading control (mean ± s.e.m.). n=3-4 independents. **d**) HAP1 parental (WT) and JMY knockout (JMY KO) cells were treated with vehicle (control) or etoposide (500nM) for 6h before ChIP. qPCR was performed on ChIP chromatin with results expressed as fold over IgG (mouse non-specific IgG) after normalising to input levels. Graphs (fold + SD) show p53 recruitment to the promoter of *BAX* (i) or *CDKN1A* (ii). Representative experiment shown, n=2 independent experiments. * p < 0.05, ** p < 0.01, *** p < 0.0001, Student’s t-test.

**Figure S4. JMY-deficiency increases DNA damage**. **a)** i) MCF7 cells transfected with JMY or non-targeting siRNA for 72h and treated with vehicle (control), etoposide (10µM) or 4NQO (100nM) for the last 16h before performing Comet assays. Quantification of the comet DNA content distributed between the head (black) and tail (grey) (ii) or the tail length (iii), n=3 independent experiments (mean ± s.e.m.). * p < 0.01 Student’s t-test. **e)** U2OS cells expressing FLAG-NLS-hJMY (FLAG). JMY was detected using anti-FLAG antibody and DAPI was used to visualise the nuclear DNA. Scale bars = 10µm (a) and 40µm (d).

**Figure S5. The absence of JMY delays the DNA damage response. a)** i) U2OS cells transfected with JMY or non-targeting siRNA for 72h and treated with vehicle (control) or etoposide (50µM) for the last 2h before immunofluorescence. ii) Quantification of γH2AX foci per cell; graph represents the mean number of foci per cell ± s.e.m, n=4 independent experiments each with N=>100 cells per condition. **b)** U2OS cells were transfected as in **a**) and treated with vehicle (control) or etoposide (50µM) for the last 6h. **c**) HAP1 parental (WT) and JMY knockout (JMY KO) cells were treated with either vehicle (control) or etoposide (500nM) for 6h. For **b)** and **c)** i) γH2AX protein expression and ii) quantification of γH2AX levels after normalising for GAPDH (n=3 independent experiments, representative experiment shown). **d)** i) U2OS cells were transfected as in **a**) and treated with etoposide (50μM) for the indicated time points before immunofluorescence with phospho-ATM/ATR substrate antibody. ii) Graph represents relative fluorescence (mean ± s.e.m.), n=3 independent experiments each with N=>100 cells per condition. **e)** i) HAP1 parental (WT) and JMY knockout (JMY KO) cells were treated with vehicle (control) or etoposide (500nM) for 16h before immunofluorescence with phospho-ATM/ATR substrate antibody. ii) Graph represents relative fluorescence (mean ± s.e.m.) as described in **d**), n=3 independent experiments each with N=>100 cells per condition. * p < 0.05, Student’s t-test. Scale bar = 10µm.

**Figure S6. Overexpression of nuclear JMY reduces the accumulation of DNA lesions. a)** and **b)** Western blot of cell extracts from U2OS cells stably expressing nuclear JMY derivatives and untranfected (-) controls. Ectopic JMY was detected with anti-HA antibody and endogenous and ectopic JMY is detected by anti-JMY antibody. ii) Graphs represent quantification of ectopic JMY as fold change over endogenous JMY expression (-) after normalising with GAPDH. **c)** i) U2OS cells stably expressing nuclear wild-type mouse JMY (NLS-mJMY) or vector control were treated with vehicle (control) or etoposide (10μM) for 16h before performing Comet assays. Quantification of the DNA content distributed between the head (black) and tail (grey) (ii) and tail length (iii), n=4 independent experiments (mean ± s.e.m.). * p < 0.01 and ** p < 0.001, Student’s t-test. Scale bar = 40µm.

**Figure S7. JMY ablation reduces cell proliferation and increases sensitivity to chemotherapeutic agents. a)** and **c)** Proliferation curves of HAP1 parental (WT) and JMY knockout (JMY KO) cells treated with vehicle (control) or etoposide (100nM) (**a**) and ATM (ATMi; KU60019, 500nM), ATR (ATRi; AZD6738, 500nM) or DNA-PK (DNA-PKi; M3814, 1μM) inhibitors (**b**) as indicated. Graphs represent cell confluence as fold change after normalising to time zero images (mean ± SD), n=3 independent experiments, representative experiment shown. **b)** and **d)** HAP1 parental (WT) and JMY knockout (JMY KO) cells were treated with vehicle (control), etoposide (as indicated) or 4NQO (100nM) (**c**), or ATM (ATMi; 500nM), ATR (ATRi; 500nM) and DNA-PK (DNA-PKi; 1μM) inhibitors (**d**) in the presence or absence of etoposide (100nM) before collecting and analysing by flow cytometry. Graphs represent percentage subG1 (mean ± s.e.m.), n=3-6 independent experiments. **e)** HAP1 parental (WT) and JMY knockout (JMY KO) cells were treated with vehicle (control), etoposide (as indicated) or 4NQO (100nM), before collecting and analysing apoptosis by flow cytometry. Graphs represent percentage of cells undergoing early (black) or late (grey) apoptosis (mean ± s.e.m.), n=3 independent experiments. * p < 0.05, ** p < 0.01, *** p < 0.001 and **** p < 0.0001, Student’s t-test.

**Figure S8. Uncropped western blots.**

**Table S1. Enriched pathways influenced by JMY depletion during the DNA damage response.** Selected enriched KEGG and REACTOME pathways obtained from the g:GO analysis from gProfiler. Significant threshold was set as FDR (q-value < 0.05).

**Table S2. p53-downstream targets influenced by JMY depletion.** Differentially expressed p53-dependent genes influenced by the lack of JMY during the DNA damage response (q-value < 0.05). The list was curated based on Fischer M. database ^1^. FC: Fold-change.

**Table S3. List of antibodies.**

**Table S4. List of primers.**

**Supplementary References:**

1. Fischer, M. Census and evaluation of p53 target genes. *Oncogene* **36**, 3943-3956, doi:10.1038/onc.2016.502 (2017).
